# Cytotoxicity of CD56^bright^ NK Cells towards Autologous Activated CD4^+^ T Cells Is Mediated through NKG2D, LFA-1 and TRAIL and Dampened via CD94/NKG2A

**DOI:** 10.1371/journal.pone.0031959

**Published:** 2012-02-22

**Authors:** Natasja Nielsen, Niels Ødum, Birgitte Ursø, Lewis L. Lanier, Pieter Spee

**Affiliations:** 1 Translational Immunology, Novo Nordisk A/S, Måløv, Denmark; 2 Monocyte Biology, Inflammation Biology, Novo Nordisk A/S, Måløv, Denmark; 3 Department of Biology, Faculty of Science, University of Copenhagen, Copenhagen, Denmark; 4 Department of Microbiology and Immunology and the Cancer Research Institute, University of California San Francisco, San Francisco, California, United States of America; Karolinska Institutet, Sweden

## Abstract

In mouse models of chronic inflammatory diseases, Natural Killer (NK) cells can play an immunoregulatory role by eliminating chronically activated leukocytes. Indirect evidence suggests that NK cells may also be immunoregulatory in humans. Two subsets of human NK cells can be phenotypically distinguished as CD16^+^CD56^dim^ and CD16^dim/−^CD56^bright^. An expansion in the CD56^bright^ NK cell subset has been associated with clinical responses to therapy in various autoimmune diseases, suggesting an immunoregulatory role for this subset *in vivo*. Here we compared the regulation of activated human CD4^+^ T cells by CD56^dim^ and CD56^bright^ autologous NK cells *in vitro*. Both subsets efficiently killed activated, but not resting, CD4^+^ T cells. The activating receptor NKG2D, as well as the integrin LFA-1 and the TRAIL pathway, played important roles in this process. Degranulation by NK cells towards activated CD4^+^ T cells was enhanced by IL-2, IL-15, IL-12+IL-18 and IFN-α. Interestingly, IL-7 and IL-21 stimulated degranulation by CD56^bright^ NK cells but not by CD56^dim^ NK cells. NK cell killing of activated CD4^+^ T cells was suppressed by HLA-E on CD4^+^ T cells, as blocking the interaction between HLA-E and the inhibitory CD94/NKG2A NK cell receptor enhanced NK cell degranulation. This study provides new insight into CD56^dim^ and CD56^bright^ NK cell-mediated elimination of activated autologous CD4^+^ T cells, which potentially may provide an opportunity for therapeutic treatment of chronic inflammation.

## Introduction

Natural killer (NK) cells are a critical component of the innate immune response, and were initially described as lymphoid cells that exhibit a “natural cytotoxicity” against virally infected cells and tumor cells without prior activation [1], [2]. The ability of NK cells to distinguish between potential target cells and healthy cells is regulated by an integration of signals derived from a complex repertoire of activating and inhibitory receptors [3]. Activating NK cell receptors, such as NKG2D and the natural cytotoxicity receptors (NCR), typically recognize ligands that are stress-induced, pathogen-derived or tumor-associated. In contrast, inhibitory receptors such as the inhibitory killer cell immunoglobulin-like receptors (iKIR) and the CD94/NKG2A heterodimer, monitor the absence of MHC class I proteins, which are normally expressed on healthy cells but can be downregulated upon infection or cellular stress. This ability to detect downregulation of MHC class I molecules is referred to as “missing-self” detection [4].

Studies have shown that depletion of NK cells can strongly exacerbate disease in mouse models of chronic inflammation, including myelin oligodendrocyte glycoprotein (MOG)-induced experimental autoimmune encephalomyelitis (EAE) [5], a model of multiple sclerosis (MS), as well as Staphylococcus aureus- and collagen-induced arthritis (CIA) [6], [7]. NK cell immunoregulatory activity was perforin-dependent in both a CD4^+^ T cell transfer model of colitis [8] as well as in EAE [9] and CIA [7], suggesting that NK cell cytolytic activity plays an important immunoregulatory role *in vivo*. Recent studies in different EAE models have shown that activated CD4+ T cells are protected from NK cell lysis by their expression of Qa-1, the mouse homolog of the non-classical MHC class Ib molecule HLA-E, which binds the inhibitory receptor CD94/NKG2A on NK cells. Interruption of the interaction between Qa-1 and CD94/NKG2A through a genetic mutation, or administration of a blocking anti-NKG2A F(ab)'2 fragment, resulted in potent NK cell-mediated elimination of pathogenic CD4^+^ T cells and amelioration of EAE [9], and was associated with decreased infiltration of MOG-specific T cells and reduced microglia activation in the central nervous system [10]. Furthermore, a recent study has shown that NK cells ameliorate CIA by lysis of two subsets of disease-inducing CD4^+^ T cells: follicular helper (Tfh) and Th17 cells. Blocking with an anti-NKG2A F(ab)'2 fragment lead to enhanced lysis of Tfh and Th17 cells by NK cells, and disease arrest [7]. These studies suggest that NK cells can regulate established immune responses both locally, at inflammatory sites, as well as systemically, in lymphoid organs.

Human NK cells comprise approximately 10% of all peripheral blood lymphocytes and can be divided into two subsets based on surface expression of CD56 (NCAM) and CD16 (FcγRIII). In peripheral blood, approximately 90% of circulating NK cells are CD56^dim^CD16^+^, whereas the remaining 10% are CD56^bright^CD16^dim/−^ [11]. In contrast, CD56^bright^ NK cells are abundant in secondary lymphoid organs (particularly in tonsils and lymph nodes), and NK cells with a CD56^bright^KIR^−^NKG2A^+^ phenotype accumulate in inflamed tissues, such as in synovial fluid and tissue in rheumatoid arthritis, psoriatic skin plaques, and in spinal fluid and brain lesions in multiple sclerosis [12], [13], diseases where effector CD4^+^ T cells play an important role in disease pathogenesis.

Indirect evidence suggests that human NK cells may have an immunoregulatory function in controlling activated immune cells. Patients with familial hemophagocytic lymphohistiocytosis (FHL), who suffer from impaired cell cytolytic activity, develop hyper-inflammation and abnormal activation and infiltration of macrophages and T cells [14]. In patients with relapse-remitting (RR)-MS, it has been shown that NK cell activity drops prior to clinical relapse, and that clinical remission is preceded by an increase in NK cell activity [15]. Furthermore, an expansion of CD56^bright^ NK cells correlates with clinical responses to therapy in RR-MS patients treated with IFN-β, Linomide, Zenapax or methyl-prednisolone [16], [17]. These studies imply the involvement of NK cells, and particularly the CD56^bright^ subset, in regulating chronic inflammation.

The mechanism of action of immunoregulatory NK cells in human autoimmune disease *in vivo* remains unclear. Numerous studies have demonstrated NK cell-mediated killing of activated T cells [18]–[21], dendritic cells (DCs) [22], [23], macrophages [24] and microglia [25] *in vitro*. The activating NK cell receptor NKG2D has been shown to be an important receptor stimulating NK cell-mediated elimination of activated CD4^+^ T cells *in vitro* [20], [21]. However, the role of other receptors and ligands is unclear. Furthermore, most *in vitro* studies of human NK cell-mediated cytotoxicity have not distinguished between the CD56^dim^ and CD56^bright^ subsets of NK cells, which is important in relation to understanding NK cell-mediated clearance of pathogenic cells *in vivo*, where these subsets preferentially localize in different tissues. The purpose of this study was therefore to further examine the regulation of autologous activated CD4^+^ T cells by CD56^dim^ and CD56^bright^ NK cells.

## Results

### Activated NK cells degranulate in response to activated, but not resting, autologous CD4^+^ T cells

**Figure 1:**
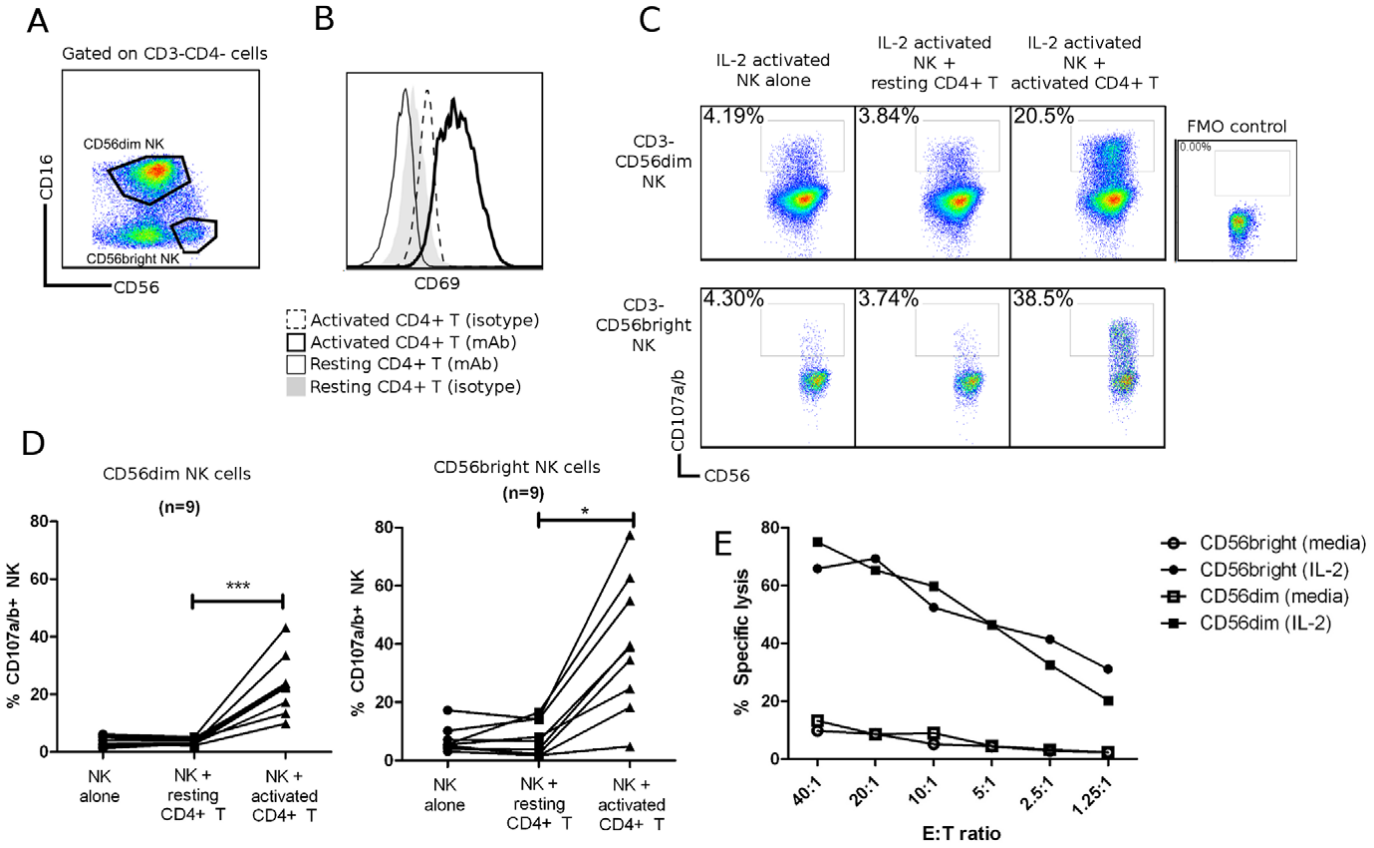
Activated NK cells kill activated, but not resting, CD4^+^ T cells. (A) Representative gating strategy. NK cells were defined as viable, CD3^−^ singlets. NK cell subsets were defined based on expression of CD16 and CD56. (B) Activation of CD4^+^ T cells was confirmed by CD69 upregulation. CD4^+^ T cells were activated for 4 days with anti-CD3+anti-CD28 Dynabeads (propionate was added on day 3). Resting CD4^+^ T cells were unstimulated in media for 4 days. (C–D) NK cells were cultured for 4 days in IL-2, and CD4^+^ T cells were activated as described. Resting CD4^+^ T cells were unstimulated for 4 days in culture. Autologous NK cells and CD4^+^ T cells were co-cultured at an E∶T ratio of 1∶1 for 4 hours with FITC-conjugated anti-CD107a+anti-CD107b mAb. Flow cytometry was performed to determine CD107a/b expression on NK cell subsets. Data shown in (C) are for a representative donor, (D) are for n = 9. Data represent mean ± SEM. * *P*<0.05; *** *P*<0.001. (E) Sorted CD56^dim^ (□/▪) and CD56^bright^ (○/•) NK cells were cultured in media (□/○) or IL-2 (▪/•) for 4 days, and co-cultured with ^51^Cr-labeled activated CD4^+^ T cells in a ^51^Cr-release assay. Experiment shown is representative of n = 3.

We characterized the regulation of activated CD4^+^ T cells by NK cells. It has previously been shown that *in vitro* activated, but not resting, CD4^+^ T cells are susceptible to NK cell-mediated lysis *in vitro* [20], [21]. To further characterize this process, untouched CD4^+^ T cells and NK cells were purified from blood of healthy donors by negative selection, and were cultured separately for 4 days. NK cells were activated with 200 IU/mL IL-2. CD3^−^ CD56^dim^ and CD56^bright^ NK cell were distinguished by flow cytometry based on differential expression of CD16 and CD56, as shown in Fig. 1A (the full gating strategy is presented in Fig. S1). CD4^+^ T cells were activated with anti-CD3 and anti-CD28 Dynabeads (with the addition of 10 mM propionate on day 3), and resting CD4^+^ T cells were cultured in media alone for 4 days. Activation of CD4^+^ T cells was confirmed by upregulation of CD69, as shown in Fig. 1B for a representative donor.

After 4 days of activation, autologous CD4^+^ T cells and NK cells were co-cultured at an E∶T ratio of 1∶1 for 4 hours. NK cell degranulation was assessed by the addition of FITC-conjugated anti-CD107a (lysosomal-associated membrane protein (LAMP)-1) and anti-CD107b (LAMP-2) antibodies during co-culture, as CD107a and CD107b are expressed on the surface of cytolytic granules in association with granule exocytosis, a prerequisite for NK cell-mediated cytotoxicity. The expression of CD107a/b on the surface of cytotoxic cells is a well-validated marker for degranulation and correlates well with specific lysis of the target cell [26], [27]. As shown in Fig. 1C, both subsets of IL-2 activated NK cells (CD56^bright^ and CD56^dim^) degranulated in response to activated, but not resting, CD4^+^ T cells. The extent of degranulation varied between donors, but as shown in Fig. 1D, an average of 23%±3.4% CD56^dim^ NK cells degranulated in response to activated CD4^+^ T cells, compared to 39%±7.6% CD56^bright^ NK cells. We verified that the degranulation of NK cells corresponded with the killing of CD4^+^ T cells. Activated CD4^+^ T cells were labeled with ^51^Cr, and co-cultured with sorted CD56^dim^ or CD56^bright^ NK cells. As shown in Fig. 1E, both subsets of IL-2-activated NK cells efficiently killed activated CD4^+^ T cells, whereas resting NK cells did not kill activated CD4^+^ T cells. Activated NK cells did not kill resting CD4^+^ T cells (data not shown). This confirms that IL-2 activated CD56^dim^ and CD56^bright^ NK cells both degranulate and kill activated, but not resting, autologous CD4^+^ T cells.

### Cytokines enhance degranulation of CD56^dim^ and CD56^bright^ NK cells

**Figure 2:**
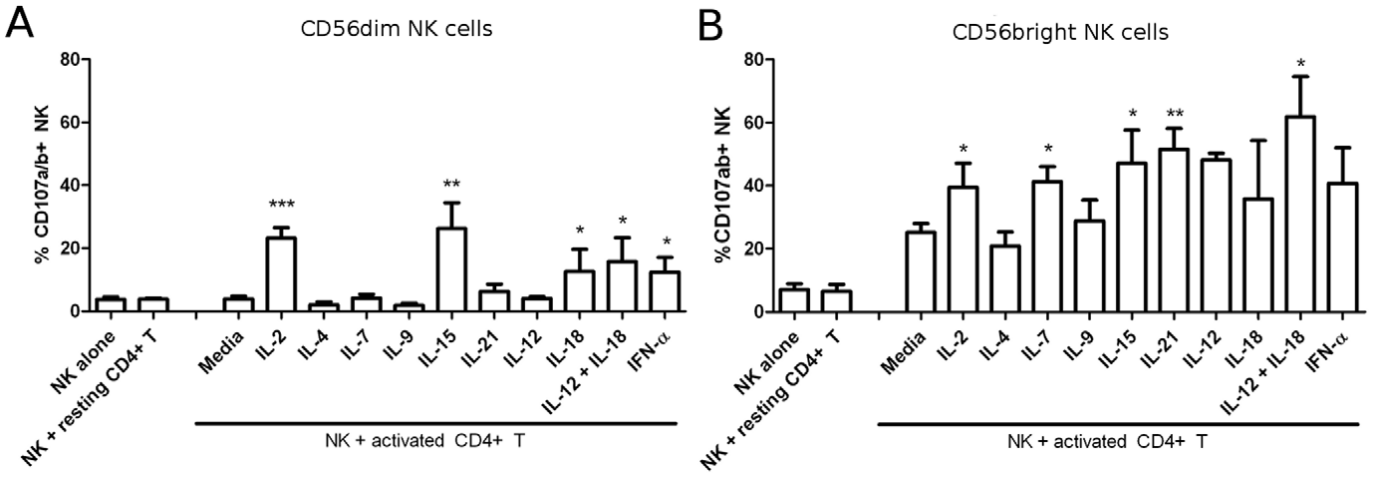
CD56^bright^ NK cells have a higher cytotoxic potential towards activated CD4^+^ T cells. Autologous NK cells and CD4^+^ T cells were isolated, and CD4^+^ T cells were activated for 4 days as described. NK cells were activated as indicated: 200 IU/mL IL-2, 25 ng/mL IL-4, 25 ng/mL IL-7, 25 ng/mL IL-9, 5 ug/mL IL-15, 100 ug/mL IL-21, 50 ug/mL IL-12, 0.25 mg/mL IL-18 or 100 U/mL IFN-αA. NK cells and CD4^+^ T cells were co-cultured for 4 hours with FITC-conjugated anti-CD107a+anti-CD107b antibodies. Flow cytometry was performed to determine degranulation of (A) CD56^dim^ NK cells and (B) CD56^bright^ NK cells. Data represent mean ± SEM of n≥4 experiments. Statistical significance is calculated in comparison to resting NK cells (media) co-cultured with activated CD4^+^ T cells. * *P*<0.05, ** *P*<0.005, *** *P*<0.001.

In a chronic inflammatory environment, such as in the synovium of an inflamed RA joint, NK cells are exposed to a complex combination of cytokines and chemokines. We therefore addressed the effect of different cytokines on NK cell-mediated cytotoxicity towards activated CD4^+^ T cells. IL-2 and IL-15 are “classical” NK stimuli, that are essential for NK cell development and homeostasis [28], and enhance NK cell proliferation, cytotoxicity and IFN-γ secretion [29], [30]. Other cytokines such as type I interferons or IL-12+IL-18 are known to promote IFN-γ production [31], proliferation [32] and Th1 polarization by NK cells [33]. Many studies on NK cell-mediated cytotoxicity *in vitro* do not distinguish between the CD56^dim^ and CD56^bright^ populations, and we therefore investigated how cytokines stimulate the two NK cell subsets in our experimental setting. As shown in Fig. 2A, CD56^dim^ NK cells only degranulated in response to activated CD4^+^ T cells when stimulated with IL-2, IL-15, IL-18, IL-12+IL-18 or IFN-α. Culture with IL-4, IL-7, IL-9, IL-12 alone or IL-21 did not lead to CD56^dim^ NK cell degranulation.

We observed that several cytokines enhanced the degranulation of CD56^bright^ NK cells towards activated CD4^+^ T cells: IL-2, IL-7, IL-15, IL-21, IL-12, IL-18, IL-12+IL-18 and IFN-α. In contrast, IL-4 and IL-9 did not affect the degranulation of CD56^bright^ NK cells towards activated CD4^+^ T cells. These results suggest that numerous cytokines stimulate the degranulation of both NK cell subsets towards activated CD4^+^ T cells: IL-2, IL-15, IL-12+IL-18 and IFN-α. Furthermore, CD56^bright^ NK cells are also activated by IL-7 and IL-21.

### NK cell degranulation towards activated CD4^+^ T cells is primarily controlled by NKG2D, NKp46, LFA-1 and NKG2A in both NK cell subsets

NK cell-mediated lysis of a target cell is a result of an integration of signals received through activating and inhibitory NK cell receptors [3]. We investigated which receptors on NK cells, and corresponding ligands on CD4^+^ T cells, are involved in the recognition and lysis of activated CD4^+^ T cells. The expression of potential ligands on activated CD4^+^ T cells was analyzed and compared to expression on CD4^+^ T cells cultured in media alone. For all ligands studied, expression on freshly isolated CD4^+^ T cells was identical to the expression found after 4 days in media (data not shown). We also studied the expression of the corresponding NK cell receptors on IL-2 activated NK cells compared to resting NK cells, and assessed whether blocking the interaction between the ligand and its corresponding receptor on NK cells affected degranulation. NK cell degranulation was not altered by the presence of an isotype-matched control Ig during co-culture, and we verified that the addition of antibodies to NK cells alone did not result in NK cell degranulation (data not shown).

**Figure 3:**
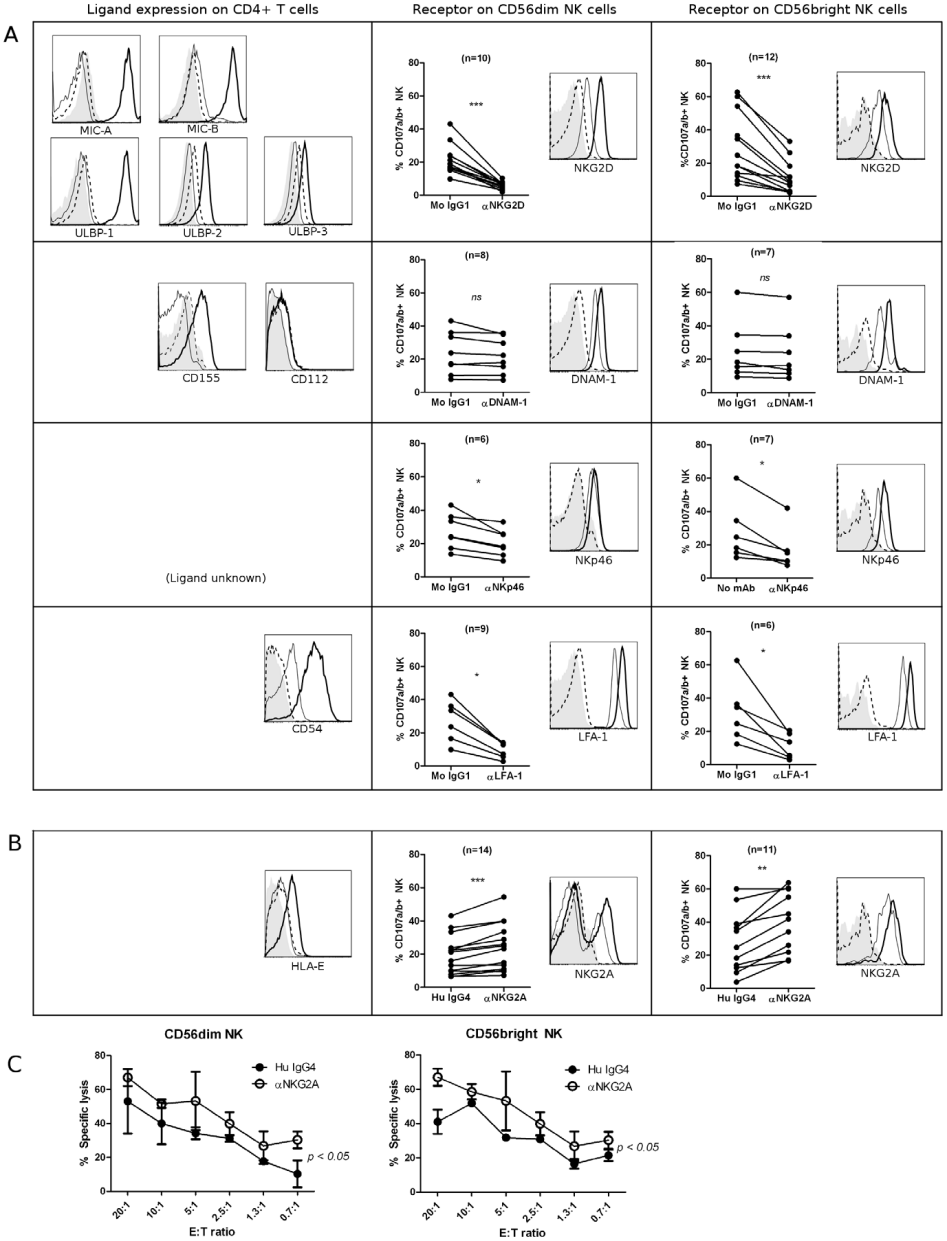
Multiple receptors and ligands are involved in NK cell-mediated lysis of activated CD4^+^ T cells. Role of (A) activating and (B) inhibitory NK receptors in NK cell degranulation. Left column: representative histograms (of n≥3) for surface expression of ligands on activated (thick black line) and resting CD4^+^ T cells (thin black line). Isotype-matched control Ig are represented by dashed line (activated CD4^+^ T) and filled histogram (resting CD4^+^ T). Middle- and right column: NK and CD4^+^ T cells were activated for 4 days *in vitro* as described, and co-cultured for 4 hours with 10 ug/mL mAb (or relevant isotype-matched control Ig). Degranulation is shown for CD56^dim^ (middle column) and CD56^bright^ (right column) NK cells. Representative histograms of surface expression of receptors on activated (thick black line) and resting NK cells (thin black line). Isotype-matched control Ig are represented by dashed line (activated NK) and filled histogram (resting NK). * *P*<0.05, ** *P*<0.005, *** *P*<0.001. (C) Sorted IL-2-activated CD56^dim^ and CD56^bright^ NK cells were co-cultured with ^51^Cr-labeled activated CD4^+^ T cells in a ^51^Cr-release assay with human IgG4 isotype control (•) or anti-NKG2A mAb (○). Data represents n = 3 experiments.

As shown in Fig. 3A, the activating receptor NKG2D plays a major role in NK cell-mediated lysis of activated CD4^+^ T cells. It has previously been shown that activated CD4^+^ T cells upregulate NKG2D ligands and become susceptible to NK cell-mediated lysis via NKG2D [20], [21]. As shown in Fig. 3A for a representative donor, we confirmed that activated CD4^+^ T cells express high levels of the NKG2D ligands MIC-A, MIC-B and ULBP-1. The addition of an anti-NKG2D neutralizing antibody reduced degranulation of CD56^dim^ and CD56 ^bright^ NK cells by 70%±2.7% and 63.4%±5.2%, respectively, relative to an isotype-matched control Ig. This confirms that NKG2D is an important activating receptor in NK cell-mediated lysis of autologous activated CD4^+^ T cells.

We found that activated CD4^+^ T cells upregulate CD155 (poliovirus receptor, PVR), a ligand for the activating NK cell receptor DNAM-1. We could not detect expression of CD112 (poliovirus-related receptor 2, PRR2), another DNAM-1 ligand, on CD4^+^ T cells. Blocking DNAM-1, however, did not affect degranulation of either NK cell subset towards activated CD4^+^ T cells (Fig. 3A). It has been shown that two additional NK receptors can interact with CD155: TIGIT, an inhibitory receptor expressed by all NK cells [34], and CD96 (TACTILE), which promotes NK cell adhesion to target cells expressing CD155 [35]. Despite the expression of CD155 on activated CD4^+^ T cells, blocking CD96 or TIGIT did not affect the degranulation of either subset of NK cells (Fig. S2A).

Adding a neutralizing anti-NKp46 antibody to the co-culture significantly reduced degranulation of CD56^dim^ NK cells by 25.3%±3.6%, and of CD56^bright^ NK cells by 38.2%±6.4% (Fig. 3A). Blocking the NCRs NKp30 or NKp44 did not affect degranulation (Fig. S2B). Although we detected CD48, the ligand for the activating NK cell receptor 2B4, on both resting and activated CD4^+^ T cells, we could not find a role for 2B4 in NK cell killing of activated CD4^+^ T cells (Fig. S2B).

Efficient cytolytic function of NK cells and cytotoxic CD8^+^ T cells is dependent on adhesion of the β_2_ integrin LFA-1 to CD54 (intercellular adhesion molecule 1, ICAM-1) on target cells [36]. Resting CD4^+^ T cells constitutively express CD54 (Fig. 3A), and activated CD4^+^ T cells further upregulate CD54 expression. Blocking LFA-1 in our co-culture significantly reduced degranulation of CD56^dim^ NK cells by 66.6%±1.7%, and of CD56^bright^ NK cells by 65.4%±7.5%.

Resting CD4^+^ T cells express low levels of the non-classical MHC class I molecule HLA-E (Fig. 3B), which binds the inhibitory CD94/NKG2A receptor on NK cells, whereas activated CD4^+^ T cells upregulate HLA-E expression. Blocking the interaction between HLA-E and the inhibitory receptor CD94/NKG2A with an anti-NKG2A mAb resulted in an average 31.3%±5.3% increase in the percentage of degranulating CD56^dim^ NK cells, and an almost two-fold (96.6%±36.9%) increase in degranulation of CD56^bright^ NK cells. The inhibitory receptor CD94/NKG2A thus plays a major role in NK cell-mediated lysis of activated CD4^+^ T cells. Although we found that activated CD4^+^ T cells upregulate expression of HLA-G, a ligand for the inhibitory NK cell receptor LIR-1, blocking LIR-1 did not affect the degranulation of NK cells (Fig. S2C).

**Figure 4:**
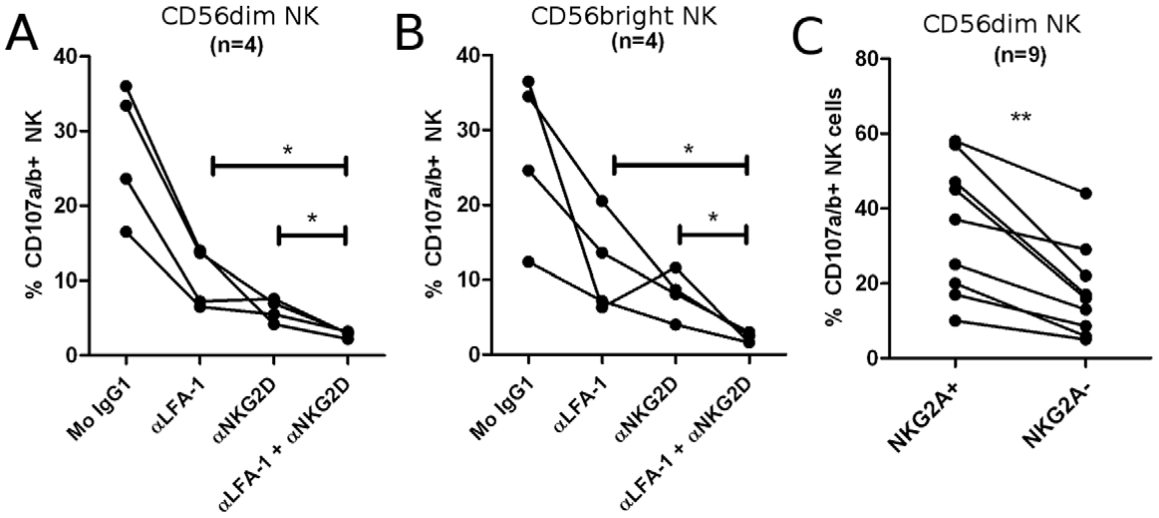
NKG2D and LFA-1 act synergistically in mediating NK cell degranulation. NK and CD4^+^ T cells were activated for 4 days *in vitro* as described, and co-cultured for 4 hours with FITC conjugated anti-CD107a+anti-CD107b mAbs. 10 ug/mL blocking antibodies (or relevant isotype-matched control Ig) were added where indicated. Flow cytometry was performed to assess degranulation of (A) CD56^dim^ and (B) CD56^bright^ NK cells. (C) CD56^dim^ NK cells were gated based on surface expression of CD94/NKG2A, and degranulation of each subset was evaluated. * *P*<0.05.

It has previously been shown that simultaneous engagement of NKG2D, 2B4 and LFA-1 on NK cells defines a minimum requirement for target cell lysis by resting NK cells [37], and that these receptors act synergistically in mediating NK cell cytotoxicity. As shown in Fig. 4A and 4B for CD56^dim^ and CD56^bright^ NK cells, respectively, we found that LFA-1 and NKG2D act synergistically in inducing NK cell degranulation, as blocking both receptors completely inhibited the degranulation of NK cells.

CD56^dim^ NK cells express the inhibitory CD94/NKG2A receptor in a heterogeneous, stochastic manner, and we investigated whether NK cells expressing CD94/NKG2A preferentially degranulate in response to activated CD4^+^ T cells. As shown in Fig. 4C, we found that an average of 35.1%±5.9% CD56^dim^NKG2A^+^ NK cells degranulate, compared to 17.8%±4.2% CD56^dim^NKG2A^−^ NK cells. A higher level of cytotoxic function among NKG2A^+^ NK cells, compared to NKG2A^−^ NK cells, has been observed previously [9], [38]. Such an analysis cannot be carried out on CD56^bright^ NK cells as these are all NKG2A^+^.

**Figure 5:**
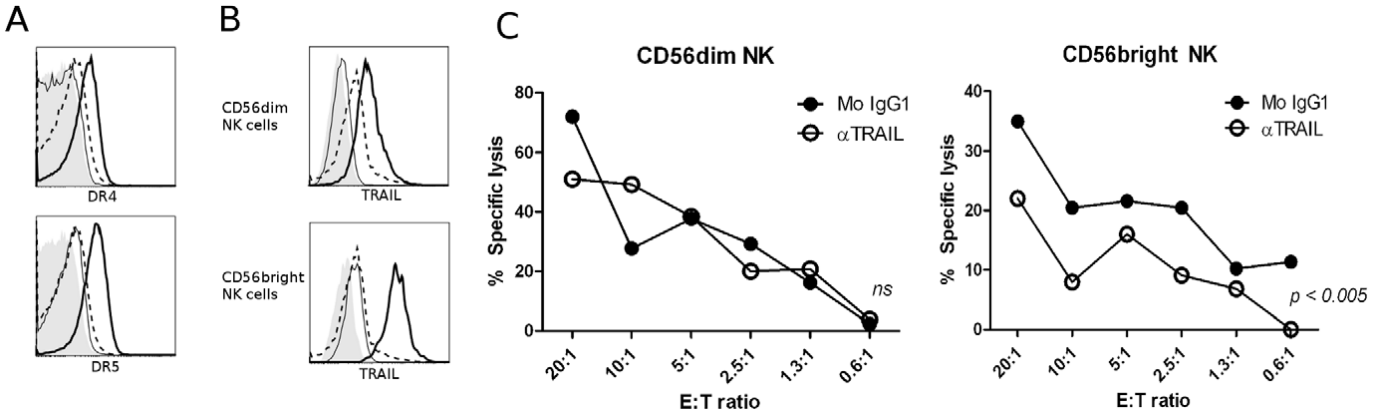
NK cells mediate TRAIL-dependent cytotoxicity towards activated CD4^+^ T cells. Autologous NK cells and CD4^+^ T cells were isolated and activated for 4 days as described. Resting NK and CD4^+^ T cells were unstimulated in media for 4 days. (A) Representative histograms for flow cytometric analysis of surface expression of TRAIL-R1 (DR4) and TRAIL-R2 (DR5) on activated CD4^+^ T cells (thick black line) and resting CD4^+^ T cells (thin black line). Isotype-matched control Ig are represented by dashed line (activated CD4^+^ T) and filled histogram (resting CD4^+^). (B) Histograms are representative of TRAIL surface expression on activated NK cells (thick black line) and resting NK cells (thin black line). Isotype-matched control Ig are represented by dashed line (activated NK) and filled histogram (resting NK). (C) Sorted IL-2-activated CD56^dim^ and CD56^bright^ NK cells were co-cultured with ^51^Cr-labeled activated CD4^+^ T cells in a ^51^Cr-release assay with isotype-matched control Ig (•) or anti-TRAIL mAb (○). Experiment shown is representative of n = 3.

NK cells are able to kill target cells by means other than perforin-mediated cytotoxicity, and we addressed whether NK cells eliminate activated CD4^+^ T cells via the TNF-related apoptosis-inducing ligand (TRAIL) pathway, as this pathway has been implicated in NK cell-mediated elimination of immune cells both *in vivo* and *in vitro* [39], [40]. TRAIL can interact with five death receptors (DR), three of which are decoy receptors (DR1, DR2 and DR3) that block TRAIL-induced apoptosis, and two (DR4 and DR5) that induce apoptosis in target cells [40]. As shown in Fig. 5A, we found that activated, but not resting, CD4^+^ T cells express both DR4 and DR5. We confirmed that both subsets of NK cells express TRAIL after activation with IL-2 (Fig. 5B), and TRAIL expression is highest in the CD56^bright^ subset. As shown in Fig. 5C, blocking TRAIL with a neutralizing antibody significantly inhibited killing of activated CD4^+^ T cells by CD56^bright^ NK cells, but had no effect on killing by CD56^dim^ NK cells, relative to an isotype-matched control Ig. This suggests a role for TRAIL, albeit minor, in CD56^bright^ NK cell-mediated killing of autologous activated CD4^+^ T cells *in vitro*.

## Discussion

It is becoming increasingly appreciated that NK cells may play an immunoregulatory role in limiting autoimmune responses. Elimination of chronically activated immune cells is one mechanism by which NK cells perform this immunoregulatory role, and in this study we have demonstrated that activated NK cells degranulate and kill activated, but not resting, autologous CD4^+^ T cells. We found that both subsets of human NK cells, CD56^dim^ and CD56^bright^, kill activated CD4^+^ T cells. Although CD56^dim^ NK cells are typically cited as being the more cytotoxic subset due to higher levels of perforin, granzymes and cytolytic granules [41], other studies have shown that following activation, CD56^bright^ NK cells are equally, if not more, cytotoxic [42], [43]. Our study confirms that both subsets are equally cytotoxic following IL-2 activation.

In a chronic inflammatory environment, NK cells will be exposed to a plethora of cytokines, and we studied the effect of different cytokines on NK cell degranulation. IL-2 and IL-15 are classical NK cell stimuli, and are essential for NK cell development and homeostasis [28]. These cytokines, as well as type I interferons (IFN-α) and IL-12+IL-18, are known to enhance NK cell proliferation, cytotoxicity and IFN-γ secretion [29]–[33]. We found that IL-2, IL-15, IL-12+IL-18 and IFN-α enhanced degranulation of both subsets of NK cells towards activated CD4^+^ T cells. Furthermore, we found that IL-7 and IL-21 stimulated degranulation of CD56^bright^, but not CD56^dim^, NK cells. The IL-7 receptor (IL-7Rα) is only expressed by CD56^bright^ NK cells [30], and IL-7 promotes survival and enhances cytotoxicity of these cells [44]. The IL-21 receptor is expressed by all NK cells in the blood, and IL-21 stimulates NK cell survival, cytotoxicity and IFN-γ secretion [30]. The synovial fluid of RA patients has been found to contain high levels of IL-12 [45], IL-18 [46], IL-15 [47], IL-7 [48] and IL-21 [49], and cytokines such as IL-12, IL-18 and IFN-α are produced by activated DCs and macrophages and have also been implicated in chronic inflammation [30], making it plausible that NK cells, in a chronic inflammatory setting, can become activated by several cytokines. It is interesting to highlight that regardless of how NK cells were activated, the same activating and inhibitory receptors controlled degranulation of NK cells in response to activated autologous CD4^+^ T cells (data not shown).

We found that NKG2D, LFA-1 and NKp46 are involved in NK cell degranulation towards activated CD4^+^ T cells in both CD56^dim^ and CD56^bright^ NK cells. We detected high levels of several NKG2D ligands on activated CD4^+^ T cells, and numerous studies have shown that T cells upregulate single or numerous NKG2D ligands and become susceptible to NKG2D-mediated lysis following activation with various stimuli *in vitro* [20], [21], [50], [51]. Discrepancies as to which NKG2D ligands are upregulated, as well as expression levels, are likely a result of how CD4^+^ T cells are activated as well as the time frame for analysis, as we found that ligand expression varied between donors but was highest after 4 days (data not shown). In addition, *MICA* and *MICB* genes are highly polymorophic [52], so different allelic variants may behave differently. NKG2D has also been implicated as an important receptor in NK cell-mediated lysis of macrophages [24], tumor cells [53] and microglia [25] as well as regulatory T cells [19].

We show that the lysis of activated CD4^+^ T cells by NK cells is dependent on engagement of the β_2_ integrin LFA-1. Adhesion through LFA-1 is a requirement of efficient target cell lysis by NK cells and cytotoxic CD8^+^ T cells [36], and engagement of LFA-1 not only mediates adhesion, but can also activate NK cells [54]. We found that NKG2D and LFA-1 act synergistically in mediating degranulation, as it was necessary to block both receptors simultaneously to completely inhibit degranulation of NK cells. Simultaneous engagement of NKG2D, 2B4 and LFA-1 has previously been shown to define a minimum requirement for target cell lysis by resting NK cells [37].

The activating receptor DNAM-1 has been implicated in NK cell-mediated lysis of DCs [22] and myeloma cells [53]. Despite the expression of one ligand for DNAM-1 on activated CD4^+^ T cells (CD155 (poliovirus receptor (PVR)), but not CD112 (Nectin-2)), blocking DNAM-1 did not affect degranulation of NK cells. CD155 has also been shown to interact with the NK cell receptors TIGIT (an inhibitory receptor that binds CD155 with a higher affinity than DNAM-1) [34] and CD96 (TACTILE, which promotes NK cell adhesion to target cells expressing CD155) [35]. However, blocking CD96 or TIGIT did not affect degranulation of either NK cell subset. A previous study has shown that monocyte-derived reactive oxygen species (ROS) is required for optimal expression of CD155 on activated T cells [55], and the expression of CD155 on activated CD4^+^ T cells in our experiments may thus be below a threshold necessary to induce a signal via DNAM-1, CD96 or TIGIT.

NK cells can also induce target cell death by membrane-bound and secreted TNF-related apoptosis-inducing ligand (TRAIL), which transduces apoptotic signals upon binding death receptors DR4 (TRAIL-R1) and DR5 (TRAIL-R2) [40]. We found that activated CD4^+^ T cells upregulate both DR4 and DR5 and become susceptible to TRAIL-mediated lysis. It has been shown that NK cells eliminate immature DCs *in vivo* via TRAIL [39], and our studies indicate a minor role for TRAIL in CD56^bright^ NK cell-mediated killing of autologous activated CD4^+^ T cells *in vitro*.

Expression of the non-classical MHC class I molecule HLA-E has been shown to protect DCs [38] and CD4^+^ T cells [18] from NK cell-mediated lysis by interactions with the inhibitory receptor CD94/NKG2A. We found that activated CD4^+^ T cells upregulate HLA-E and were protected from NK cell-mediated lysis, as the addition of an anti-NKG2A antibody significantly enhanced killing of activated CD4^+^ T cells. Blocking NKG2A had a greater effect on enhancing degranulation of CD56^bright^ NK cells, which is not surprising as this subset constitutively expresses higher levels of NKG2A, whereas only approximately half of CD56^dim^ NK cells express NKG2A, and at a lower density.

It is interesting why healthy T cells, upon activation, transiently upregulate ligands for NK cell receptors that render them susceptible to NK cell-mediated lysis. It has been proposed that NK cells, during the course of a normal immune response, reduce potentially excessive inflammation by eliminating some activated immune cells [56]. The susceptibility of a cell to NK cell-mediated lysis depends on a balance between ligands expressed for activating and inhibitory NK cell receptors. Interestingly, resting DCs and microglia are susceptible to NK cell-mediated cytotoxicity [57], [58], whereas activation of these cells upregulates MHC class I molecules and confers resistance to lysis [25]. On the contrary, resting monocytes, monocyte-derived macrophages and T cells are protected from NK cell-mediated cytotoxicity, but upon activation these cells upregulate NKG2D ligands and become susceptible to NK cell lysis [9], [18], [25], [59], [60]. We found that resting CD4^+^ T cells expressed much lower levels of all MHC class I molecules compared to activated CD4^+^ T cells (Fig. S3), yet resting CD4^+^ T cells were not susceptible to NK cell-mediated lysis, and blocking with an anti-HLA class I antibody did not result in NK cell degranulation. The lack of activating receptor ligands likely explains why resting CD4^+^ T cells are protected from NK cell cytotoxicity, regardless of expression of MHC class I molecules. The specificity of NKG2D ligand expression to activated CD4^+^ T cells highlights why unleashing NK cells by masking an inhibitory receptor such as NKG2A presents an interesting therapeutic opportunity to treat chronic inflammation compared to current strategies, as it may allow for lysis of a small subpopulation of activated, potentially pathogenic CD4^+^ T cells, while leaving the remaining T cell repertoire intact. Indeed, in a mouse model of RA, CIA, it has recently been shown that blocking NKG2A specifically leads to NK cell-mediated lysis of pathogenic Tfh and Th17 cells, and thus abrogation of disease development [7].

Our finding that CD56^bright^ NK cells readily degranulate and kill activated CD4^+^ T cells has interesting therapeutic implications, as NK cells with a CD56^bright^ phenotype have been implicated in numerous autoimmune diseases. CD56^bright^ NK cells have been associated with clinical remission in MS [15], and MS patients responding to treatment with IFN-β showed an expansion in CD56^bright^ NK cells and a concurrent reduction in CD4^+^ and CD8^+^ T cells [16], [17]. Furthermore, reduced brain inflammation following treatment of MS patients with Daclizumab (an antagonistic humanized mAb against the IL-2Rα chain) correlated with an expansion of CD56^bright^ NK cells and a reduction in CD4^+^ and CD8^+^ T cells *in vivo* [61]. Similar results were found in patients with autoimmune uveitis who were treated with Daclizumab [62]. In a study of relapsing-remitting (RR)-MS, a reduction in NK cell functional activity correlated with the appearance of active lesions and pre-ceded clinical attacks, suggesting that NK cells may play a protective role in ongoing MS [63].

CD56^bright^ NK cells are found in abundance at inflammatory sites in a number of diseases [12], [13], [64]–[67], and an important question that arises is to what extent CD56^bright^ NK cells in blood are comparable to CD56^bright^ NK cells found at inflammatory sites. Phenotypical characterization of CD56^bright^ NK cells from the synovial fluid of RA patients has shown that synovial fluid CD56^bright^ NK cells share many features with CD56^bright^ NK cells from the blood: they are NKG2A^+^CD25^+^KIR^−^ and produce IFN-γ in response to low concentrations of cytokines [68]. In contrast to CD56^bright^ NK cells in the blood, however, they are CD69^+^ and NKp44^+^ and therefore have an activated phenotype, suggesting local activation [69]. The accumulation of CD56^bright^ NK cells at inflammatory sites may be due to preferential recruitment of this subset from the blood, an idea that is supported by the finding that CD56^bright^ NK cells in the blood constitutively express CCR5, CCR7 and CD62L, which could regulate their recruitment to inflammatory sites and lymphoid tissues [70], [71]. CD56^bright^ NK cells express CCR5, which potentially regulates their homing to inflammatory sites in RA where high concentrations of MIP-1α, MIP-1β and RANTES have been found [65]. It cannot be ruled out that the presence of NK cells with a CD56^bright^ phenotype in inflammatory sites might also be due to local proliferation or local differentiation.

Our finding that both subsets of activated NK cells degranulate in response to activated CD4^+^ T cells, and use the same receptors in doing so regardless of how they are activated, suggests that any NK cell that receives activation signals that are sufficient to overcome the threshold required for activation, has the potential to kill a target cell. Enhancing NK cell-mediated clearance of autoreactive CD4^+^ T cells in chronic inflammation by masking an inhibitory receptor such as NKG2A, therefore, presents an interesting therapeutic possibility. Indeed, it has been shown in a mouse model of MS, MOG-induced EAE, that administration of an anti-NKG2A F(ab)'2 fragment results in potent NK cell-mediated elimination of infiltrating pathogenic CD4^+^ T cells and microglia, and amelioration of EAE [9], [10]. The advantage of this approach is the specific targeting of activated, potentially autoreactive, immune cells, and not healthy tissues. Further studies are required to elucidate the mechanism of action of immunoregulatory NK cells in chronic inflammation *in vivo*, and how the cytotoxic potential of these regulatory immune cells can be utilized to treat autoimmunity.

## Materials and Methods

### NK cell and CD4^+^ T cell isolation and in vitro culture

Peripheral blood mononuclear cells (PBMC) were obtained from Histopaque (Sigma-Aldrich) separation of buffy coats from healthy donors supplied anonymously from the Clinical Immunology Blood Bank, The State University Hospital, Copenhagen. Some donors were healthy adult volunteers at UCSF whose PBMC were obtained by density gradient centrifugation over Ficoll-Paque (GE Healthcare). All volunteers gave informed consent to participate in the study (approved by University of California San Francisco Committee on Human Research IRB 10-00265). NK cells and CD4^+^ T cells were isolated from PBMC by negative selection using antibody-coated bead separation (EasySep Enrichment Kits, StemCell Technologies) and cultured for 4 days at 1×10^6^ cells/mL in RPMI-1640 GlutaMAX™ medium (Invitrogen) supplemented with 10% fetal calf serum and 1% penicillin and streptomycin. Cell purities were 95%–99% (confirmed by flow cytometry). Isolated CD4^+^ T cells were activated with anti-CD3+anti-CD28 DynaBeads (Invitrogen) for 4 days; 10 mM sodium propionate (Sigma-Aldrich) was added for the last 24 hours of culture. NK cells were cultured for 4 days in media containing 200 U/mL recombinant human IL-2 (Proleukin, Novartis). Alternatively, NK cells were stimulated with the following recombinant human cytokines where indicated: 25 ng/ml IL-7, IL-4, IL-9; 50 ug/mL IL-12, 0.25 mg/mL IL-18 (all from R&D Systems); 100 ug/mL IL-21 (Miltenyi Biotec), 5 ug/mL IL-15 (PeproTech), 100 U/mL IFN-αA (PBL InterferonSource). After 4 days in culture, viability of NK and CD4^+^ T cells (both resting and activated) was typically more than 90%. For some experiments, NK cells were isolated from PBMC using antibody-coated bead separation (Miltenyi Biotec).

### Cytotoxicity assays

Following 4 days of activation, autologous NK cells and CD4^+^ T cells were co-cultured at an effector∶target (E∶T) ratio of 1∶1 for 4 hours at 37°C with FITC-conjugated anti-CD107a mAb (H4A3, BD Biosciences), FITC-conjugated anti-CD107b mAb (H4B4, BD Biosciences) and GolgiStop™ containing monensin (BD Biosciences). The following anti-human mAbs were added at 10 ug/mL where indicated: NKG2D (149810, R&D Systems), NKG2A (NNC0141-0100, Novo Nordisk A/S), LIR-1 (GHI/75, BioLegend), 2B4 (C1.7, BioLegend), DNAM-1 (DX11, BioLegend), NKp30 (P30-15, BioLegend), NKp44 (P44-8, BioLegend), NKp46 (9E2, BioLegend), LFA-1/CD11a (HI111, BioLegend), TRAIL (RIK-2, BioLegend), TIGIT (MBSA43, eBioscience) and CD96 (NK92.39, BioLegend). Mouse IgG1 (R&D Systems) and human IgG4 (Sigma-Aldrich) were used as isotype-matched Ig controls. None of the antibodies resulted in CD107a/b expression on the cell surface when added to NK cells alone. For ^51^Cr-release assays, CD4^+^ T cells were labeled with Na^51^Cr0_4_ (Amersham Biosciences). NK cells were added at effector∶target (E∶T) ratios from 40∶1 to 0.625∶1 in duplicates. Cells were incubated for 5 hours at 37°C, and released ^51^Cr was measured in a TopCount (PerkinElmer). Spontaneous lysis was determined by incubating target cells alone, and maximum lysis was determined by the addition of 10% Triton X-100 in PBS to target cells. Percentage specific lysis = (experimental ^51^Cr release−spontaneous ^51^Cr release)/(maximum ^51^Cr release−spontaneous ^51^Cr release)×100.

### Flow cytometry

Following the 4-hour cytotoxicity assay, cells were stained with PerCP-conjugated anti-CD3 (SK7, BD Biosciences), PerCP-conjugated anti-CD4 (11830, R&D Systems), Pacific Blue-conjugated anti-CD16 (3G8, BD Biosciences), PE-conjugated anti-NKG2A (Z199, Beckman Coulter), Alexa Fluor 700-conjugated anti-CD56 (B159, BD Biosciences) and Near-IR LIVE/DEAD® Fixable Dead Cell Stain (Invitrogen). NK cells were gated as viable, CD3^−^CD4^−^ cells and NK cell subsets were defined based on expression of CD56 and CD16.

CD4^+^ T cells were surface stained for the presence of antigens with the following antibodies at 10 ug/mL or 1∶100 dilution: anti-HLA-E (3D12HLA-E, eBioscience), anti-MHC class I (DX17, BD Biosciences), PE-conjugated anti-CD48 (BJ40, BioLegend), anti-MIC-A (159213), anti-MIC-B (236511), anti-ULBP-1 (170818, R&D Systems), anti-ULBP-2 (165903, R&D Systems), anti-ULBP-3 (166510, R&D Systems), anti-CD112 (R2.525, BD Biosciences), PE-conjugated anti-CD155 (SKII.4, BioLegend), APC-conjugated anti-CD54 (HA58, BD Biosciences), anti-TRAIL-R1 (DJR1, BioLegend) and anti-TRAIL-R2 (DJR2-4(7-8), BioLegend). Unconjugated antibodies were detected by subsequent incubation with APC-conjugated donkey anti-mouse IgG (Jackson ImmunoResearch). Mouse IgG1, mouse IgG2a and mouse IgG2b were used as isotype-matched controls (R&D Systems). CD4^+^ T cells were stained with Pacific Blue-conjugated anti-CD3 (OKT3, eBioscience), Alexa Fluor 700-conjugated anti-CD4 (BioLegend), PerCP-conjugated anti-CD8 (SK1, BD Biosciences) and Near-IR LIVE/DEAD® Fixable Dead Cell Stain (Invitrogen). CD4^+^ T cells were gated as viable, CD3^+^CD4^+^CD8^−^ cells. All flow cytometry was carried out using a BD LSR II flow cytometer.

### Statistical analysis

Statistical significances were determined by using the paired, two-tailed Student's t-test, or a two-way ANOVA. All calculated averages were defined as parametric mean ± SEM. Statistical significance is defined as the following: * *P*<0.05; ** *P*<0.01; *** *P*<0.001. Statistics were determined with GraphPad Prism Software.

## Supporting Information

Figure S1 **Representative gating strategy used to define CD56^dim^ and CD56^bright^ NK cells.**
(TIFF)

Figure S2 **CD96, TIGIT, NKp30, NKp44, 2B4 and LIR-1 are not involved in NK cell killing.** (A, C) Left column: representative histograms for surface expression of ligands on activated (thick black line) and resting CD4^+^ T cells (thin black line). Isotype-matched control Ig are represented by dashed line (activated CD4^+^ T) and filled histogram (resting CD4^+^). Flow cytometry was performed to assess degranulation of CD56^dim^ (middle column) and CD56^bright^ (right column) NK cells. Representative histograms show surface expression of receptors on activated NK cells (thick black line) and resting NK cells (thin black line). Isotype-matched control Ig are represented by dashed line (activated NK) and filled histogram (resting NK). * *P*<0.05 (B) Sorted IL-2-activated CD56^dim^ and CD56^bright^ NK cells were co-cultured with ^51^Cr-labeled activated CD4^+^ T cells in a ^51^Cr-release assay, with relevant isotype-matched control Ig or anti-2B4 mAb.
(TIFF)

Figure S3 **CD4^+^ T cell expression of HLA class I.** Representative histograms for surface expression of HLA class I on activated (thick black line) and resting CD4^+^ T cells (thin black line). Isotype-matched control Ig are represented by dashed line (activated CD4^+^ T) and filled histogram (resting CD4^+^).
(TIFF)
