# Supplementary figures and images for: Cytotoxicity of CD56bright NK Cells towards Autologous Activated CD4+ T Cells Is Mediated through NKG2D, LFA-1 and TRAIL and Dampened via CD94/NKG2A
Source: PLoS One. 2012 Feb 22;7(2):e31959. doi: 10.1371/journal.pone.0031959 (PMC3284517; doi:10.1371/journal.pone.0031959)

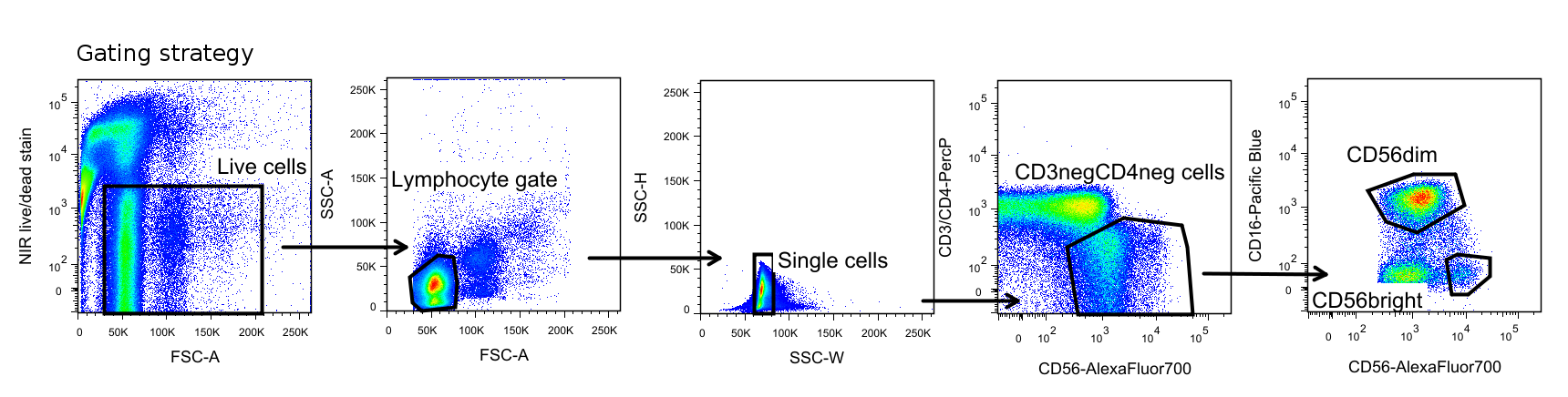

Supplement: Figure S1 — Representative gating strategy used to define CD56dim and CD56bright NK cells. (TIFF) [file pone.0031959.s001.tiff]

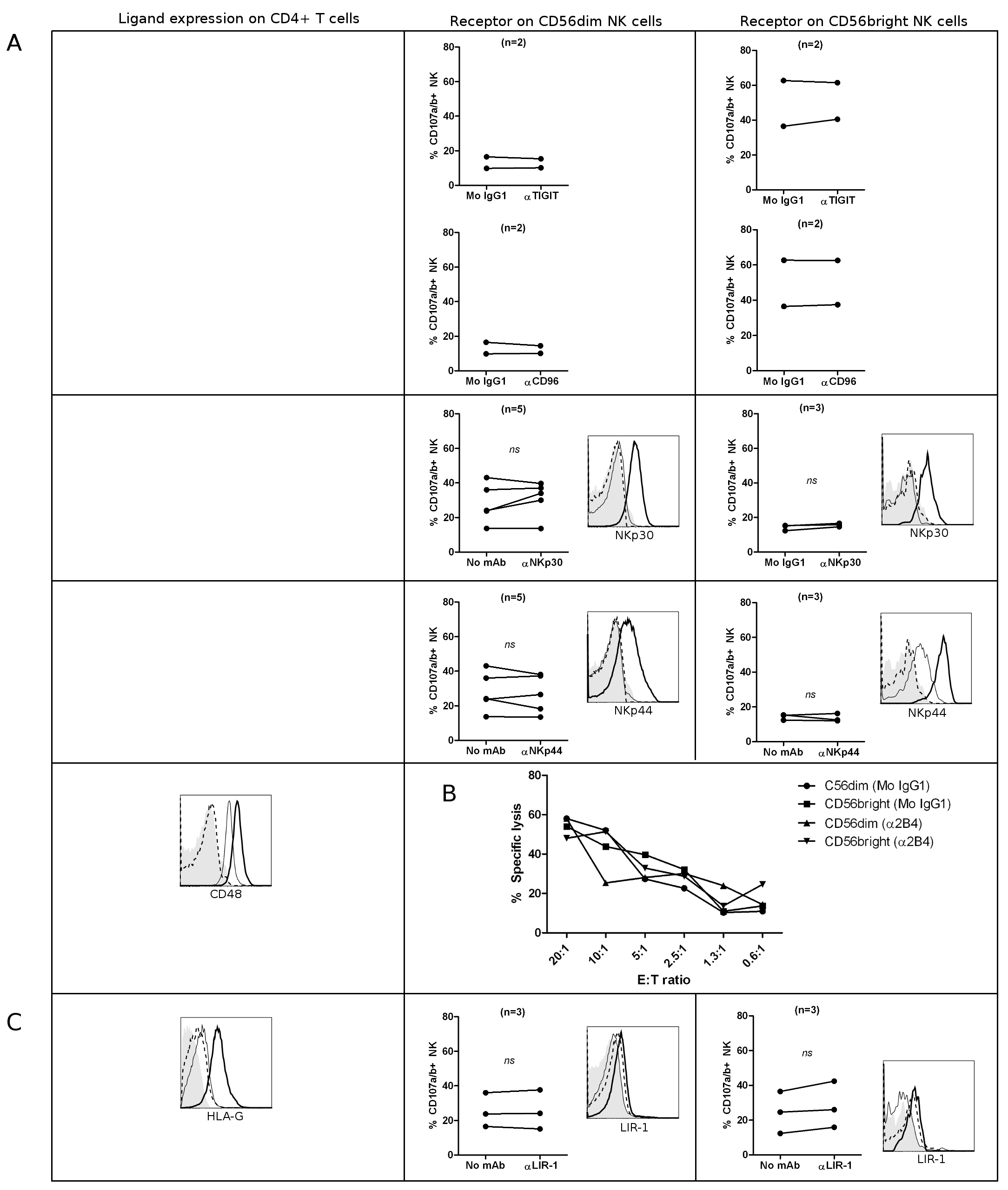

Supplement: Figure S2 — CD96, TIGIT, NKp30, NKp44, 2B4 and LIR-1 are not involved in NK cell killing. (A, C) Left column: representative histograms for surface expression of ligands on activated (thick black line) and resting CD4+ T cells (thin black line). Isotype-matched control Ig are represented by dashed line (activated CD4+ T) and filled histogram (resting CD4+). Flow cytometry was performed to assess degranulation of CD56dim (middle column) and CD56bright (right column) NK cells. Representative histograms show surface expression of receptors on activated NK cells (thick black line) and resting NK cells (thin black line). Isotype-matched control Ig are represented by dashed line (activated NK) and filled histogram (resting NK). * P<0.05 (B) Sorted IL-2-activated CD56dim and CD56bright NK cells were co-cultured with 51Cr-labeled activated CD4+ T cells in a 51Cr-release assay, with relevant isotype-matched control Ig or anti-2B4 mAb. (TIFF) [file pone.0031959.s002.tiff]

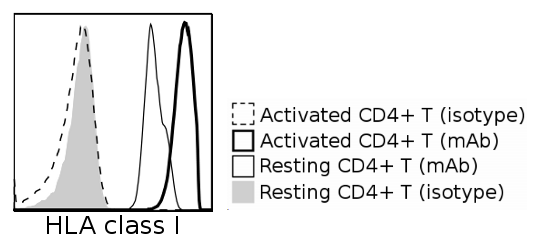

Supplement: Figure S3 — CD4+ T cell expression of HLA class I. Representative histograms for surface expression of HLA class I on activated (thick black line) and resting CD4+ T cells (thin black line). Isotype-matched control Ig are represented by dashed line (activated CD4+ T) and filled histogram (resting CD4+). (TIFF) [file pone.0031959.s003.tiff]
